# The association between hemoglobin level and sarcopenia in Chinese patients with Crohn’s disease

**DOI:** 10.1186/s12876-024-03182-2

**Published:** 2024-03-04

**Authors:** Nandong Hu, Jingjing Liu, Xifa Gao, Hongye Tang, Jiangchuan Wang, Zicheng Wei, Zhongqiu Wang, Xiaoli Yu, Xiao Chen

**Affiliations:** 1 Department of Radiology, Affiliated Hospital of Nanjing University of Chinese Medicine, 155 Hanzhong road, 210029 Nanjing, China; 2 Department of Radiology, Funan County People’s Hospital, 36 santa road, 236300 Fuyang, Anhui China

**Keywords:** Albumin, Crohn’s disease, Hemoglobin, Montreal classification, Sarcopenia

## Abstract

Sarcopenia and anemia are common complications in patients with Crohn’s Disease (CD). However, few studies have shown the association between sarcopenia and hemoglobin levels in CD patients. This retrospective study aimed to explore such association in Chinese patients with CD. Two hundred and twelve adult CD inpatients who underwent computed tomography (CT) or magnetic resonance imaging (MRI) examinations from July 2019 to December 2021 were included in the study. Sarcopenia was defined according to the cutoff value of skeletal muscle index of lumbar spine 3 (SMI-L3) (< 44.77cm^2^/m^2^ for males and < 32.5cm^2^/m^2^ for females). The CD patients were divided into two groups based on the presence or absence of sarcopenia. Clinical data, hemoglobin levels, and other laboratory data were retrospectively collected. The association between hemoglobin levels and sarcopenia was analyzed by univariable and multivariable logistic regression analysis. Sarcopenia occurred in 114 CD patients (53.8%). Compared to patients without sarcopenia, patients with sarcopenia had a lower proportion of L1 (30.7% vs. 45.8%, *p* = 0.032) and B1 classification (58.8% vs. 72.4%, *p* = 0.037). Patients with sarcopenia had significantly lower levels of hemoglobin (Hb) (116.5 ± 22.8 vs. 128.1 ± 21.0, *p* < 0.001). The prevalence of sarcopenia increased with the decrease in hemoglobin level (p for trend < 0.05). Linear regression analysis showed that hemoglobin levels were associated with SMI-L3 (β = 0.091, *p* = 0.001). Multivariable logistic regression analysis found that higher hemoglobin levels (OR:0.944; 95% CI: 0.947,0.998; *p* = 0.036) were independent protective factors for sarcopenia. Lower hemoglobin levels are independently associated factors of sarcopenia in adult Chinese patients with CD.

## Introduction

Primary sarcopenia is an age-related muscle failure characterized by loss of muscle mass, strength, and/or low physical function [1]. Although sarcopenia is commonly associated with aging, it can also occur in patients with chronic wasting diseases, such as inflammatory bowel diseases (IBD), malignancy, chronic obstructive pulmonary disease, chronic liver and kidney disease, congestive heart failure, and patients who are chronically bedridden or receiving steroid therapy [2]. Mechanistically, the reduced muscle synthesis may be related to decreased insulin-like growth factor 1, elevated pro-inflammatory cytokines, and increased oxidative stress leading to diminished AKT signaling [3, 4]. However, little is known about the exact molecular mechanism of sarcopenia [5].

Crohn’s disease (CD) is a type of inflammatory bowel disease (IBD). Over the last decade, body composition changes, including changes in fat, bone loss, osteoporosis, and sarcopenia, could occur in patients with CD [6]. Patients with CD often have poor nutritional status [7] and are at risk of malnutrition due to an imbalance between nutritional requirements and caloric loss caused by the catabolic state, especially in patients with an active inflammatory condition. Combined with various factors, such as a persistent inflammatory state and malnutrition, the incidence of sarcopenia is high in CD patients [8, 9]. At present, few studies show the associated factors with the development of sarcopenia in patients with CD. In addition, the available findings are somewhat controversial. A few studies suggested that the occurrence of sarcopenia may be related to gender, and Lee et al. [10] reported a higher incidence of sarcopenia in male CD patients, while Galata et al. [11] found the opposite results. Some studies concluded that there were no significant differences in gender, age, Montreal location, behavioral typing, disease activity index, and inflammatory marker between patients with and without combined sarcopenia [12]. However, other studies have shown that C-reactive protein (CRP) and lesion location (L2 type) are associated with the development of sarcopenia [13, 14].

Anemia or low hemoglobin level is common in patients with CD. Moreover, few recent studies showed that anemia was associated with sarcopenia [15, 16]. However, to our knowledge, no study has reported such association in patients with CD. Muscle health in CD patients is associated with treatment response [17] and surgical outcome [18]. Identifying associated factors may aid for CD management or CD-related sarcopenia clinical intervention. Therefore, we investigated the association between hemoglobin levels and sarcopenia in Chinese patients with CD.

## Materials and methods

### Study participants

**Fig. 1:**
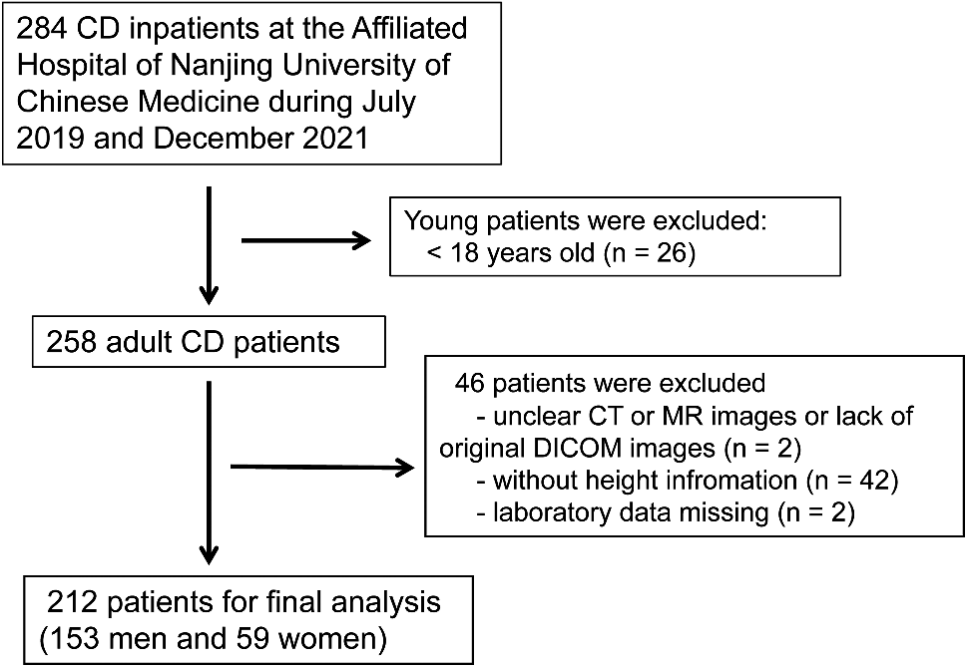
The flow chart of study

This study retrospectively collected 284 CD patients who were hospitalized with small bowel CT enterography (CTE) or small bowel magnetic resonance enterography (MRE) at the Affiliated Hospital of Nanjing University of Chinese Medicine between July 2019 and December 2021. The clinical and imaging characteristics of CD patients were recorded. Finally, 212 adult newly diagnosed CD patients with complete height information were included, comprising 153 males and 59 females, The flowchart is shown in Fig. 1. The study was approved by the ethics committee of Affiliated Hospital of Nanjing University of Chinese Medicine (2021-NL02).

The inclusion criteria were as follows: (1) meeting the diagnostic criteria of Crohn’s disease (clinical symptoms, endoscopic examinations, and imaging findings); (2) abdominal CT/MR or CTE/MRE images were clear to meet the measurement requirements; (3) imaging examinations within 1 month of performing laboratory tests such as routine blood, biochemistry, blood sedimentation, CRP or fecal calprotectin, coagulation function and endoscopy. Exclusion criteria: (1) aged < 18 years; (2) unclear CT or MR images or lack of original CT/MR images; (3) pregnant or breastfeeding; (4) incomplete information on laboratory tests such as routine blood, biochemistry, sedimentation, CRP or fecal calprotectin; (5) chronic kidney disease, chronic obstructive pulmonary disease, malignancy, congestive heart failure, chronic obstructive pulmonary, neuromuscular disease, activity limitation or prolonged braking; (6) lack of height information.

### Data collection

The following data were retrospectively collected to determine the associated factors of sarcopenia in patients with CD: (1) General information, including sex, age, height, time from symptom presentation to diagnosis, marital history, smoking, alcohol habits, granuloma detection by endoscopic biopsy, hospital days and hospital costs. (2) laboratory tests: including red blood cell count, mean red blood cell volume, white blood cell count, platelet count, lymphocyte count, neutrophils, lymphocytes, monocytes, eosinophils, basophils, hemoglobin, hematocrit, erythrocyte sedimentation rate (ESR), CRP, and fecal calprotectin, serum total protein, serum albumin, globulin, albumin to globulin ratio, prealbumin, glucose, aspartate aminotransferase, alanine aminotransferase, prothrombin time (PT), activated partial thromboplastin time (APTT), activated partial thromboplastin ratio, fibrinogen (FIB), prothrombin time (PT), D-dimer (D-D) and fibrin (pro) degradation product (FDP) and stool occult blood. Serum albumin < 35 g/L was used to define malnutrition [19, 20].

### CT or MR imaging

**Fig. 2:**
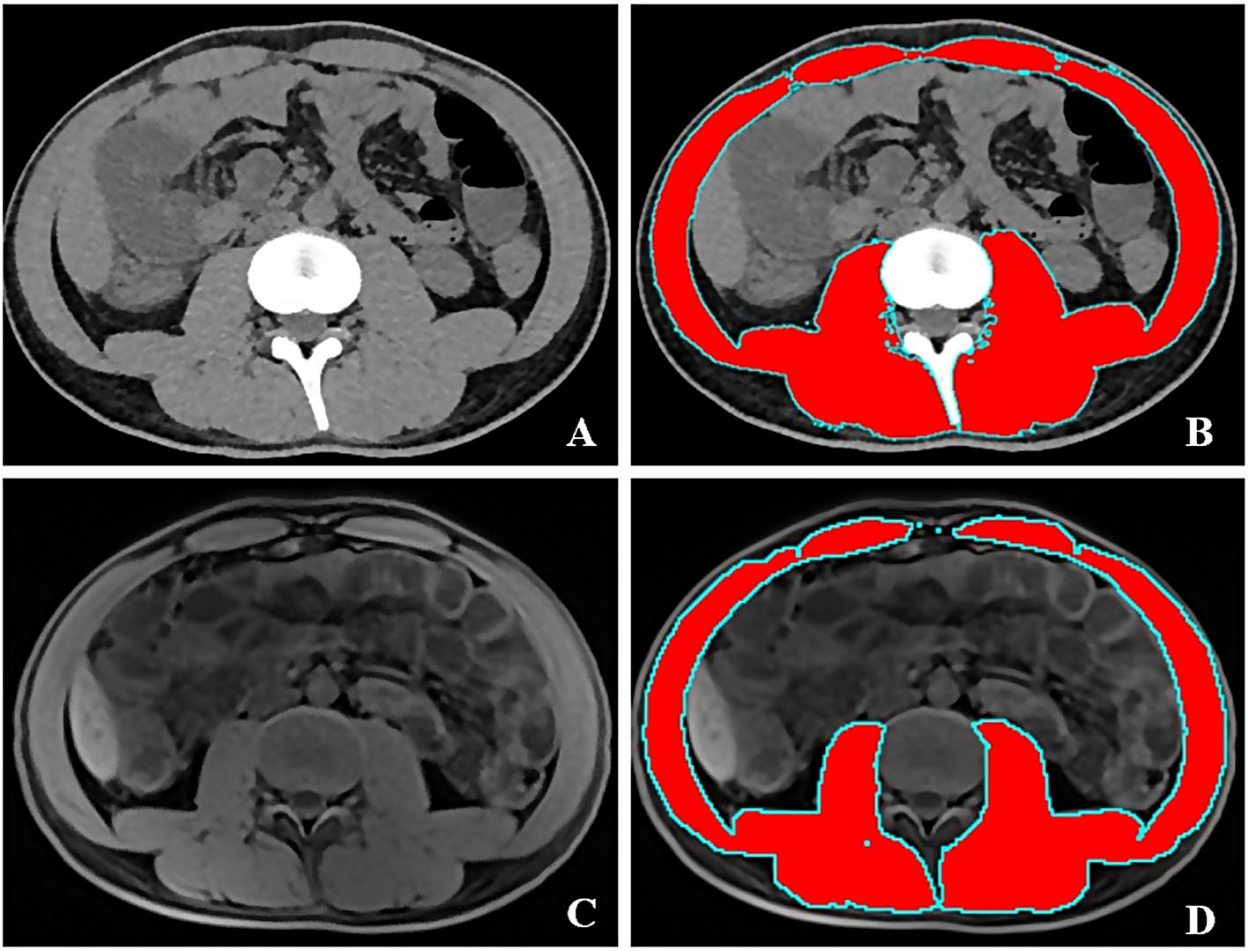
The illustration of muscle area measurement on computed tomography (CT) and magnetic resonance (MR) images. **A**, **C**: the CT and MR images; **B**, **D**: the segmentation of muscle using ImageJ software

The intestinal wall thickness of the most severely affected bowel segment, lesion location (L1 type), disease behavior (B1 type), presence of perianal lesions (P), and subcutaneous fat thickness around the navel (SFT) were evaluated on CT or MR images. Abdominal circumference (AC), and skeletal muscle area at the lumbar three vertebral level (CSA-L3) were measured using ImageJ, an image analysis software developed by the National Institutes of Health [21]. When using this software, the identification criteria for CT images of skeletal muscle were − 29 to + 150 HU. For MRI images, muscle area was measured on T1-weighted imaging and identified based on the in tissue signal differences. An illustration of muscle area measurement on CT and MR images is shown in Fig. 2. CTE/MRE evaluation: images were evaluated and measured by two senior physicians who independently observed the images on picture archiving and communication system and were blinded to the results of patient’s colonoscopy results, tests, and clinically relevant information.

### Definition of sarcopenia

The CSA-L3 was the sum of the areas of several muscles, including the psoas major, erector spinae, multifidus, lumbar square, transverse abdominis, internal oblique, external oblique, and rectus abdominis. The skeletal muscle index (SMI-L3) was calculated by CSA-L3 divided by height squared. Sarcopenia in CD patients was defined by SMI-L3 < 44.77 cm^2^/m^2^ in men and SMI-L3 < 32.5 cm^2^/m^2^ in women based on a previous study in the Chinese population [22].

### Statistical analysis

CD patients were divided into two groups based on the sex-specific SMI-L3 cut-off values: sarcopenia and non-sarcopenia groups. Continuous variables with normal or near-normal distribution were shown as mean ± standard deviation and were compared using Student t-test or one-way ANOVA; continuous variables with severely skewed distribution were shown as median (interquartile range) and data were compared using Wilcoxon rank sum test. The Shapiro-Wilk test was used to test the normal distribution of continuous data. Categorical data were present as frequencies (percentages) and comparisons between groups were made using the chi-square test or Fisher’s exact probability method. Linear regression analysis was used to assess the correlation between SMI-L3 and vertebral CT values, adiposity parameters, and laboratory indices. Univariable logistic regression was used to analyze the relationship between sarcopenia and gender, age, Crohn’s disease Montreal typing, granuloma detection, and laboratory examination indexes. Variables with *p* < 0.05 were entered to multivariable logistic regression analysis to identify independent associated factors of sarcopenia. Hemoglobin levels were transformed into categorical variables by quartiles, and linear trend tests were performed by the Linear-by-Linear Association. All statistical tests were two-tailed and *p* < 0.05 was considered statistically significant. IBM SPSS software (Version 22.0, Chicago, IL, USA) was used for statistical analysis.

## Results

### General information

One hundred and fourteen patients had sarcopenia. The characteristics of CD patients with and without sarcopenia are shown in Table 1. No significant differences were found in Montreal type between patients with and without sarcopenia. However, the occurrence of simple ileal type (L1 type) in patients with sarcopenia was significantly lower than in patients without sarcopenia (30.7% vs. 45.8%, *p* = 0.032). A similar result was observed for the non-stenotic non-penetrating type (B1 type) (58.8% vs. 72.4%, *p* = 0.037). The prevalence of malnutrition in patients with sarcopenia was significantly higher than in patients without sarcopenia (27.19% vs. 11.2%, *p* = 0.003).

**Table 1:** Clinical data in CD patients with and without sarcopenia

Variables	Sarcopenia (n = 114)	Non-sarcopenia (n = 98)	P-value
Sex			
Male, n (%)	81 (71.1%)	72 (73.5%)	0.295
Age*	27 (21,34.5)	29 (21.75,34.5)	0.311
Symptom-to-diagnosis time (month)*	12 (3,36)	12 (6,24)	0.482
Marital history (unmarried)	57 (50.9%)	38 (38.8%)	0.078
Smoking (yes)	7 (6.2%)	4 (4.1%)	0.491
Alcohol consumption (yes)	3 (2.7%)	6 (6.1%)	0.310
Fecal occult blood (positive)	61 (61.6%)	38 (47.5%)	0.059
Granuloma detection rate*	27 (25%)	31 (34.8%)	0.05
Montreal Fractal			
Age (A2)	95 (83.3%)	79 (80.6%)	0.607
Location (L1/L2/L3)	35/11/68	44/5/49	0.07
L1 type	35 (30.7%)	44 (45.8%)	0.032
Behavioral (B1/B2/B3)	67/39/8	71/22/5	0.186
B1 type	67 (58.8%)	71 (72.4%)	0.037
Perianal lesions	38 (33.3%)	44 (45.8%)	0.067
Biological agent use	85 (74.6%)	66 (67.3%)	0.247
Diabetes	1 (0.88%)	1 (1.02%)	1.00
Chronic kidney disease	0	0	/
Malnutrition	31 (27.19%)	11 (11.22%)	0.003

*Continuous variables with severe skewed distribution are reported as median (interquartile range) and data are compared between groups using Wilcoxon rank sum test. Categorical data were reported as frequencies (percentages) and comparisons between groups are made using the chi-square test

### Laboratory tests

Fecal calprotectin (*p* = 0.001), ESR (*p* = 0.003), and CRP levels (*p* < 0.001) were significantly higher in patients with sarcopenia than in patients without sarcopenia. Hemoglobin (*p* < 0.001), mean hemoglobin content (*p* < 0.001) and mean hemoglobin concentration (*p* = 0.002) levels were significantly lower and platelets (*p* = 0.008) were significantly higher in CD patients with sarcopenia than those without sarcopenia.

Regarding coagulation parameters (Table 2), patients with sarcopenia had significantly higher levels of fibrinogen and fibrin degradation products, fibrinogen to albumin ratio (FAR) (*p* < 0.001), and slightly higher levels of PT, international normalized rate, PT activity, and D-dimer than patients without sarcopenia (*p* < 0.05). Serum total protein levels (*p* = 0.001), albumin levels (*p* < 0.001), albumin ratio (*p* < 0.001), and prealbumin level (*p* < 0.001) in patients with sarcopenia were significantly lower than patients without sarcopenia.

**Table 2:** Laboratory tests in CD patients with and without sarcopenia

Variables	Sarcopenia (n = 114)	Non-sarcopenia group (n = 98)	P-value
FC (µg/g)	948.9 (658.9,1279)	685.5 (291.9,1036.6)	0.001
ESR (mm/h)	35 (18,57)	23 (12,39)	0.003
CRP (mg/L)	24.3 (11.4,57.3)	10.3 (4.0,24.3)	< 0.001
FAR	0.123 ± 0.038	0.094 ± 0.030	< 0.001
NLR	3.48 (2.34,5.01)	3.08 (1.89,4.66)	0.094
Red blood cells (×10^12^ /L)	4.47 ± 0.59	4.65 ± 0.57	0.027
Leukocytes (×10^9^ /L)	7.72 ± 3.3	7.14 ± 2.52	0.153
Hb (g/L)	116.5 ± 22.8	128.1 ± 21.0	< 0.001
Mean hemoglobin content (pg)	25.98 ± 3.31	27.54 ± 2.99	< 0.001
Mean hemoglobin concentration (g/L)	314.61 ± 15.46	320.83 ± 13.85	0.002
Neutrophils (×10^9^ /L)	4.98 (3.46,6.37)	4.12 (3.33,6.11)	0.192
Lymphocytes (×10^9^ /L)	1.41 (1.1,1.78)	1.45 (1.08,1.97)	0.496
Monocytes (×10^9^ /L)	0.6 (0.41,0.8)	0.51 (0.4,0.7)	0.065
Eosinophils (×10^9^ /L)	0.1 (0.07,0.18)	0.11 (0.06,0.2)	0.634
Basophils (×10^9^ /L)	0.02 (0,0.035)	0.02 (0,0.04)	0.443
PT (s)	13.78 ± 0.87	13.36 ± 0.70	< 0.001
PT activity (%)	96.70 ± 13.82	104.2 ± 13.39	< 0.001
Activated partial thromboplastin time (s)	42.04 ± 5.11	41.55 ± 6.05	0.547
Activated partial thromboplastin ratio	1.215 (1.12, 353)	1.205 (1.11, 1.368)	0.831
Fibrinogen (g/L)	4.47 ± 1.08	3.83 ± 0.96	< 0.001
Thrombin time (s)	17.27 ± 1.45	17.39 ± 1.10	0.501
D-dimer (D-D) (mg/L)	0.345 (0.27, 0.55)	0.28 (0.22,0.377)	0.002
FDP (mg/L)	2.21 (1.89,3.02)	1.78 (1.473,2.355)	< 0.001
Plasma antithrombin (%)	92.43 ± 11.91	93.11 ± 11.89	0.703
Aspartate aminotransferase (U/L)	14 (10,17)	14 (12,18)	0.039
Alanine aminotransferase (U/L)	13 (8,18.25)	13 (9,19.25)	0.425
Total protein (g/L)	64.516 ± 7.730	67.987 ± 7.534	0.001
Albumin (g/L)	37.250 ± 5.816	41.329 ± 5.057	< 0.001
Globulin (g/L)	27.576 ± 5.291	26.665 ± 5.483	0.227
Albumin/globulin	1.390 ± 0.342	1.6248 ± 0.422	< 0.001
Prealbumin (mg/L)	128.92 ± 53.313	155.49 ± 52.766	< 0.001
Glucose (mmol/L)	4.28 (3.94, 4.82)	4.425 (4.07, 4.715)	0.396

Continuous variables conforming to normal distribution are compared between groups using Student t-test; Continuous variables with severe skewed distribution are reported as median (interquartile range) and data are compared between groups using Wilcoxon rank sum test

*Note* CRP = C-reactive protein; ESR = erythrocyte sedimentation rate; FAR = fibrinogen to albumin ratio; FC = fecal calprotectin; FDP: Fibrin degradation products; Hb = hemoglobin; PT: Prothrombin time; NLR = neutrophil to lymphocyte ratio

### Logistic regression analysis

The associated factors for sarcopenia were further analyzed using univariable logistic regression analysis. The parameters with statistical differences in Tables 1 and 2 were included in the univariable logistic regression analysis. The results showed that simple ileal type (L1 type), non-stenotic non-penetrating type (B1 type), higher hemoglobin level, red blood cells, mean hemoglobin concentration, PT, PT activity, total blood protein, albumin, prealbumin, CRP, fecal calprotectin were associated factors for sarcopenia in CD patients (Table 3).

**Table 3:** Associated factors for sarcopenia

Variable	Univariable logistic regression analysis		Multivariable logistic regression analysis	
	P value	OR value (95% CI)	P value	OR value (95% CI)
Gender (male/female)	0.696	1.128 (0.617–2.064)	0.257	0.469 (0.126–1.739)
Age (years)	0.155	0.983 (0.959–1.007)	0.08	0.960 (0.913–1.002)
L1-type	0.025	0.524 (0.297–0.922)	0.110	1.302 (0.534–2.176)
B1-type	0.038	0.542 (0.304–0.967)	0.222	2.375 (0.959–5.885)
Perianal lesions	0.468	0.817 (0.472–1.412)		
FC (µg/g)	0.001	1.001 (1.000-1.002)	0.051	1.001 (1.000-1.002)
ESR (mm/h)	0.002	1.021 (1.008–1.034)		
CRP (mg/L)	0.001	1.018 (1.008–1.029)	0.489	1.003 (0.994–1.013)
Hb (g/L)	< 0.001	0.976 (0.963–0.989)	0.036	0.944 (0.947–0.998)
Red blood cells (×10^12^ /L)	0.028	0.59 (0.37–0.95)		
Mean hemoglobin concentration (g/L)	0.003	0.97 (0.95–0.99)		
Aspartate aminotransferase (U/L)	0.85	1.00 (0.98–1.02)		
PT(s)	< 0.001	2.070 (1.375–3.116)	0.024	1.986 (1.094–3.606)
PT activity (%)	< 0.001	0.961 (0.939–0.983)		
Fibrinogen (g/L)	< 0.001	1.869 (1.377–2.536)	0.134	1.388 (0.846–2.277)
D-Dimer (mg/L)	0.714	1.092 (0.682–1.747)		
FDP (mg/L)	0.138	1.187 (0.946–1.489)		
Total protein (g/L)	0.002	0.941 (0.906–0.978)		
Albumin (g/L)	< 0.001	0.872 (0.824–0.923)	0.08	0.944 (0.868–1.001)
Albumin/globulin	< 0.001	0.189 (0.084–0.427)		
Prealbumin (mg/L)	0.001	0.991 (0.985–0.996)		

Total protein, prealbumin and albumin/globulin were significantly correlated to albumin (*r* > 0.6). They were not included in multivariable analysis. Similar reason was for mean hemoglobin concentration

*Note* CI = confidence interval; CRP = C-reactive protein; ESR = erythrocyte sedimentation rate; FC = fecal calprotectin; Hb = hemoglobin; FDP = Fibrinogen Degradation Products; NLR = neutrophil to lymphocyte ratio; PT = prothrombin time

The variables with *p* < 0.05 were included in multivariable regression analysis after excluding parameters with strong correlations (*r* > 0.5). Despite gender was not statistically significant in the univariable analysis, we included it in the multivariable analysis because gender may be related to other laboratory parameters. Gender, whether L1 type, B1 type, fecal calprotectin, CRP, hemoglobin, fibrinogen, PT and albumin were finally included in the multivariable logistic regression analysis. The results showed that higher hemoglobin (odds ratio (OR): 0.944; 95% confidence interval (CI): 0.947, 0.989; *p* = 0.036) was independent protective factors for sarcopenia in patients with CD, while higher PT levels (OR:1.986;95%CI:1.094,3.606; *p* = 0.023) was an independent risk factor for sarcopenia in patients with CD (Table 3).

### Multivariable linear regression analysis

Subsequently, we discovered the link between SMI-L3 and Montreal location typing (L typing), mixed perianal lesions, CRP, fibrinogen, hemoglobin level, and total serum protein levels using multiple linear regression analysis. The values of SMI-L3 were associated with gender, Montreal behavioral typing (B typing), hemoglobin, fibrinogen, and serum albumin levels (*p* < 0.05 or 0.01) (Table 4).

**Table 4:** Multivariable linear regression analysis of SMI-L3 in adult patients with Crohn’s disease

variable	β	95%CI	P value
Gender (male/female)	-8.307	-10.690 to -5.924	< 0.001
L-type	-0.497	-1.266 to 0.272	0.204
B-type	-1.667	-3.282 to -0.052	0.043
Perianal lesions	-1.234	-3.145 to 0.677	0.204
CRP (mg/L)	0.001	-0.010 to 0.012	0.860
Hb (g/L)	0.091	0.038 to 0.144	0.001
FIbrinogen (g/L)	-1.033	-2.015 to -0.051	0.039
Albumin (g/L)	0.326	0.078 to 0.574	0.010

*Note* CI = confidence interval; CRP = C-reactive protein; Hb = hemoglobin

### The association between categorical hemoglobin and prevalence of sarcopenia and SMI-L3

Next, we divided hemoglobin levels into categorical data (interquartile range) and showed the association between categorical hemoglobin level and the prevalence of sarcopenia and SMI-L3 (Table 5). SMI-L3 increased with the increasing hemoglobin levels in the total population (p for trend < 0.001), men (p for trend < 0.001), and women (p for trend = 0.046). The prevalence of sarcopenia decreased with the increase of hemoglobin levels in the total population (p for trend = 0.006), men (p for trend = 0.004), and women (p for trend = 0.014).

**Table 5:** SMI-L3 and prevalence of sarcopenia in CD patients at different quartile levels of hemoglobin

Group (Hb)	Q1	Q2	Q3	Q4	P-trend
Sarcopenia					
Total	69.1% (38/55)	60.8% (31/51)	47.2% (25/53)	37.7% (20/53)	0.006
Male	65.0% (26/40)	68.4% (26/38)	44.7% (17/38)	32.4% (12/37)	0.004
Female	65.4% (22/31)	33.3% (11/28)			0.014
SMI-L3(cm^2^/m^2^)					
Total	33.94 ± 7.56	38.84 ± 6.81	44.11 ± 6.50	47.11 ± 7.13	< 0.001
Male	41.20 ± 7.41	42.29 ± 5.56	46.04 ± 6.06	48.02 ± 7.32	< 0.001
Female	30.88 ± 5.99	33.72 ± 4.49			0.046

*Note* SMI-L3 = skeletal muscle index of lumbar spine 3

Q1 are < 118.5 g/L for men; 90 g/L for women; 107 g/L for total patients

Q2 are 118.5–133 g/L for men; 90–101 g/L for women; 107-124.5 g/L for total patients

Q3 are 133–144 g/L for men; 101–111 g/L for women; 124.5-140.8 g/L for total patients

Q4 is > 144 g/L for men; >111 g/L for women; >140.8 g/L for total population

## Discussion

CD with sarcopenia is gradually becoming a hot research topic for early screening, early intervention, and prevention of adverse clinical outcomes. Malnutrition and chronic inflammation may be the major contributors to the development of sarcopenia in CD patients, and the disease severity may also be linked to this condition. Therefore, it is critical to identify patients who are at high risk for developing sarcopenia in clinical practice. In this study, we investigated the associated factors for sarcopenia in terms of clinical indicators. Our data showed that hemoglobin levels were associated with SMI-L3 and the prevalence of sarcopenia. Our findings may be valuable for CD management and CD-related sarcopenia clinical intervention.

Measuring sarcopenia may be a challenge for CD patients due to the cost or specialized staff for administration [23]. Small bowel CTE or MRE is usually used for CD diagnosis or evaluation. Sarcopenia evaluated MRE did not cause additional cost or ionizing radiation. Measurement is easy and quick for CT or MRI technicians. CT and MRI are considered gold standards for non-invasive assessment of muscle quantity [24]. Therefore, we defined sarcopenia based on CT and MR images.

In this study, we observed that 53.8% of CD patients had sarcopenia. Our result is consistent with those published data [8, 25]. Some studies also showed the associated factors for sarcopenia in CD patients [14, 26], such as nutrition-related indicators (Low albumin level) and higher levels of inflammatory markers (CRP, ESR, and FC). However, in a retrospective cohort study that included 76 patients undergoing CD surgery, CRP and albumin levels did not differ statistically between patients with and without sarcopenia [27]. Our study also showed that albumin level and inflammation markers (CRP and FC) were associated with sarcopenia in univariable analysis. However, no such associations were observed between those factors and sarcopenia in multivariable analysis, which indicated that the contribution of those factors were lower than other factors. In addition, different populations, different disease stage and different definition of sarcopenia were used in those studies which may be the main reasons. Our result showed that PT was associated with risk of sarcopenia. An previous study demonstrated that the level of PT activity in liver cancer patients with sarcopenia was higher than those without sarcopenia [28]. However, the reason for such association is unknown.

Patients with IBD frequently have anemia and is present in about two-thirds of patients at the time of initial diagnosis [29]. Few researches focused on the potential roles of hemoglobin, a marker of anemia, in IBD populations. Vinke et al. [30] found a significant correlation between hemoglobin levels and reduced muscle mass and muscle strength in patients undergoing renal transplantation. The correlation between muscle function and sarcopenia was strong in men and people with higher basal burdens [15]. Our data showed that the prevalence of sarcopenia increased with the decrease of hemoglobin. Further, linear regression showed that hemoglobin levels were associated with SMI-L3. Moreover, multivariable regression analysis after adjusting for inflammatory biomarkers also demonstrated that low hemoglobin level was associated with sarcopenia. Those analyses all indicated that hemoglobin level was an associated factor of sarcopenia in CD patients.

How low hemoglobin affects sarcopenia is not understood. The critical role of hemoglobin is oxygen transport. It is well-known that oxygen is essential for the survival of cells. Low hemoglobin levels may result in less oxygen delivery to cells or tissue. Consequently, chronic hypoxia may affect the function or quality of tissues or organs [31] and weaken their strength and performance. A recent study also showed that low hemoglobin levels could cause poor muscle oxygenation and induce low muscle mass and strength [32]. In addition, people with anemia are prone to fatigue, which may lead to a lack of physical exercise and sarcopenia [33]. Iron deficiency affects mitochondrial metabolism and myoglobin synthesis which may impair muscle performance [34]. Moreover, hemoglobin levels and sarcopenia may have a complicated and bidirectional relationship. Low hemoglobin levels indicate poorer nutritional status. Malnutrition causes anemia and sarcopenia, and chronic sarcopenic depletion may further lower hemoglobin levels. How sarcopenia affects hemoglobin is not unknown. Furthermore, anemia or low hemoglobin may be a complication of CD. Further research is necessary to determine whether the rise in hemoglobin levels can prevent or even stop the development of sarcopenia.

Some studies have shown that sarcopenia is associated with loss of response to biological agents [17, 35, 36], poor surgical outcomes [18], and longer hospital stays in patients with IBD [37, 38]. Sarcopenia is also associated with extra-intestinal manifestations, such as reduction in bone mineral density and increased incidence of non-alcoholic fatty liver disease (NAFLD) [39]. A recent review article also indicated that sarcopenia play a pivotal role in optimizing surgical outcomes in patients with CD [18]. Currently, no specific drugs have been approved to treat sarcopenia [40]. Preventive strategies have focused on identifying important risk factors and modifying these risk factors in later life [38]. In addition, it has been shown that the treatment of anaemia could improve myocyte metabolism [41]. Our study is valuable for the clinical management of CD patients from the aspect of anemia.

This study has several limitations. First, this was a retrospective single-center study with relatively small sample size because CD is not common in China. There was selection bias and confounders could not be well controlled (such as nutritional status, physical activity or sedentary activity). The retrospective design also can not show causal associations between anemia and sarcopenia. Second, we lacked clinical markers indicating the CD severity (CD activity index or Harvey-Bradshaw index) and body mass index. Therefore, we could not control them in multivariable analysis. However, the skeletal muscle index was calculated by muscle area/ height^2^. In addition, we included fecal calprotectin in our multivariable model. Fecal calprotectin is a marker of CD disease activity [42, 43]. The correlation coefficient between FCP and Crohn’s Disease Endoscopic Index of Severity reached to 0.61 [40]. Moreover, the area under the curve reached 0.93 in predict active disease on colonoscopy [44]. Third, sarcopenia was assessed based on CT or MRI, not on dual-energy x-ray and bioelectrical impedance analysis. However, lumbar muscle cross-sectional area by CT or MRI has also been used to assess sarcopenia [45]. Finally, this study only focused on Chinese CD patients. The generalizability of our findings should be validated in other ethnic groups.

In conclusion, the prevalence of sarcopenia increased with the decrease of hemoglobin level. Hemoglobin level was positively associated with SMI-L3 and lower levels of hemoglobin were independently associated with the onset of sarcopenia in CD patients.

## Data Availability

All data generated or analyzed during this study are available from the corresponding author upon reasonable request.

## Abbreviations

AC: abdominal circumference
APTT: activated partial thromboplastin time
CD: Crohn’s Disease
CI: confidence interval
CRP: C-reactive protein
CT: computed tomography
ESR: erythrocyte sedimentation rate
FAR: fibrinogen to albumin ratio
FC: fecal calprotectin
FDP: fibrin (pro) degradation product
FIB: fibrinogen
Hb: hemoglobin
MRI: magnetic resonance imaging
NLR: neutrophil to lymphocyte ratio
OR: odds ratio
PT: prothrombin time
SFT: periumbilical subcutaneous fat thickness
