# Correction: The association between hemoglobin level and sarcopenia in Chinese patients with Crohn’s disease

**DOI:** 10.1186/s12876-024-03203-0

**Published:** 2024-03-19

**Authors:** Nandong Hu, Jingjing Liu, Xifa Gao, Hongye Tang, Jiangchuan Wang, Zicheng Wei, Zhongqiu Wang, Xiaoli Yu, Xiao Chen

**Affiliations:** 1 Department of Radiology, Affiliated Hospital of Nanjing University of Chinese Medicine, 155 Hanzhong road, 210029 Nanjing, China; 2 Department of Radiology, Funan County People’s Hospital, 36 santa road, 236300 Fuyang, Anhui China

**Correction:** ***BMC Gastroenterol*** **24, 95 (2024)**

10.1186/s12876-024-03182-2

Following publication of the original article it was reported that Xiaoli Yu was missing a designation as corresponding author. The original article has been updated to correctly identify Xiaoli Yu as a corresponding author.

