# Correction: The association between hemoglobin level and sarcopenia in Chinese patients with Crohn’s disease

**DOI:** 10.1186/s12876-024-03217-8

**Published:** 2024-04-09

**Authors:** Nandong Hu, Jingjing Liu, Xifa Gao, Hongye Tang, Jiangchuan Wang, Zicheng Wei, Zhongqiu Wang, Xiaoli Yu, Xiao Chen

**Affiliations:** 1 Department of Radiology, Affiliated Hospital of Nanjing University of Chinese Medicine, 155 Hanzhong road, 210029 Nanjing, China; 2 Department of Radiology, Funan County People’s Hospital, 36 santa road, 236300 Fuyang, Anhui China

**Correction:** ***BMC Gastroenterol*** **24, 95 (2024).**

10.1186/s12876-024-03182-2

Following publication of the original article it was reported that Table 3 was missing from the PDF version of the published article due to a typesetting mistake whereby Table 4 overlaid it.

The HTML version of the article did correctly display Table 3.

The original article has been updated to correct the PDF version of the article to include Table 3.

